# The Relationship Between Myocardial Infarction and Estrogen Use: A Literature Review

**DOI:** 10.7759/cureus.46134

**Published:** 2023-09-28

**Authors:** Ayesha Javed, Phanish C Ravi, Sarah Bilal Delvi, Iqra Faraz Hussain, Arnaldo J Acosta G., Warda Iqbal, Vamsi Krishnamaneni, Saya Alasaadi, Swetapadma Pradhan, Rishabh Vashisht, Shivani Modi

**Affiliations:** 1 Gynecology, Heart's International Hospital, Rawalpindi, PAK; 2 Medicine and Surgery, Sri Venkata Sai Medical College, Telangana, IND; 3 Internal Medicine, Vydehi Institute of Medical Sciences and Research Centre, Bangalore, IND; 4 Gynecology, Karachi Medical and Dental College, Karachi, PAK; 5 Endocrinology, Alfredo Van Grieken University Hospital, Coro, VEN; 6 Internal Medicine, Katuri Medical College and Hospital, Guntur, IND; 7 Medicine, University College Dublin, Dublin, IRL; 8 Medicine, European University Faculty of Medicine, Tbilisi, GEO; 9 Internal Medicine, Non Resident Indian (NRI) Medical College, Vijayawada, IND; 10 Internal Medicine, Albert Einstein Healthcare Network, Philadelphia, USA

**Keywords:** cvd, transdermal estrogen, estrone, estradiol, myocardial infarction

## Abstract

This thorough literature evaluation was prompted by significant research into the complex interactions between estrogen use and myocardial infarction (MI). Estrogen has fascinated researchers because of its possible cardioprotective benefits and its impact on cardiovascular health. In order to clarify the connection between estrogen use and the risk of MI, this review critically examines the body of prior evidence.

This review focuses on estrogen and its pivotal role in cardiovascular health, concentrating on lipid metabolism, vasodilation, inflammation, and endothelial function. It examines contentious data about estrogen therapy's heart-protective effects, taking into account age, initiation timing, dosage, and dosage of administration. Genetic and epigenetic influences on MI risk among estrogen users highlight intricate, personalized estrogen effects.

The conclusion summarizes the main findings and emphasizes the need for an all-encompassing strategy for initiating and managing estrogen medication. It is crucial to consider patient-specific traits and risk factors to successfully customize treatment regimens. This review sheds vital light on the potential directions for better cardiovascular treatment for postmenopausal women by shedding light on the complex link between estrogen use and myocardial infarction. The review also identifies research gaps and future objectives in this area, highlighting the demand for novel medicines and individualized strategies to improve cardiovascular outcomes.

## Introduction and background

The increased morbidity and mortality rates caused by the association between estrogen use, age, and myocardial infarction (MI) have been shown to have significant effects on global health. Men are more prone to cardiovascular disease (CVD) than women, but women appear to have more CVD postmenopause, as estrogen levels significantly decrease, causing changes in body composition, increased oxidative stress, central fat deposition, and decreased vasodilation [1].

Numerous studies have examined the impact of ovarian hormone deprivation on cardiovascular and autonomic systems in mouse models [1,2]. The findings are significant because they suggest that ovarian hormone deprivation (OVX) might impact body weight, lipid profiles, insulin sensitivity, aerobic capacity, baroreflex function, and inflammation. Aerobic training (AT) has demonstrated promise in minimizing these consequences in animal models. Resting hemodynamics, anti-inflammatory responses, and reflex modulation of the circulatory system are all positively impacted by AT. This is well demonstrated by the rise in cardiac muscle interleukin-10 (IL-10) levels [3-5].

The impact of estrogen hormones on the heart's contractile response is another clue that ovarian hormones may directly suppress the growth of cardiac beta-adrenergic receptors. This effect has been observed in postmenopausal women as well as in young rats that had their ovaries removed. Estrogen replacement medication has been shown to alter these responses [6-8]. Hormone deprivation in postmenopausal women has revealed significant consequences, including but not limited to changes in heart rate variability and fetal reflex sensitivity. Increased cardiac sympathetic activity and decreased vagal influence were established as the causes of oxidative stress, arterial stiffness, and decreased flow-mediated vasodilation [9]. The fact that the risk of stroke in women doubles during the first decade following menopause and surpasses the risk for males at comparable ages is an interesting observation. Heart failure strikes 18% of women and 8% of men between the ages of 45 and 64 within five years of the initial myocardial infarction, indicating that women are more likely than men to experience this vascular catastrophe with a worse prognosis [10].

This article summarises several studies looking into the consequences of MI and estrogen use. The authors review clinical trials that involve the effects of estrogen and MI. The study aims to advance understanding of the cardiovascular risks and benefits of estrogen therapy by attempting to elucidate the complex relationship between estrogen use and MI through an analysis of the existing data. 

## Review

We used a thorough strategy to compile pertinent material for this narrative review of therapies, conforming to accepted norms and principles for narrative reviews. Electronic databases like PubMed, PsycINFO, EMBASE, and the Cochrane Library were all thoroughly searched by our team. To find possibly neglected papers, we also manually searched reference lists from systematic reviews, meta-analyses, and important articles.

Myocardial infarction, commonly known as a heart attack, is characterized by the ischemic necrosis of cardiac myocytes resulting from the rupture of an atherosclerotic plaque. The rupture exposes the plaque content, leading to thrombosis and complete occlusion of the coronary artery, causing an imbalance between blood and oxygen demand and supply, ultimately leading to the death of cardiac cells [1]. Several risk factors contribute to the development of myocardial infarction. Age is a significant factor, with men over the age of 45 and postmenopausal women over the age of 55 being more susceptible [2,3].

Studies such as the NURSES health study and WISE study have revealed that early menopause in women, secondary to ovarian malfunction or bilateral oophorectomy, is associated with a higher risk of cardiovascular disease. The prevalence rate is estimated to be 1655 cases per 100,000 people, and projections indicate that it will increase to over 1845 cases by the year 2030 [2-5]. Eastern European nations have been experiencing the highest incidence of these conditions. Studies have revealed that the prevalence of MI varies significantly with age. In individuals below the age of 60, the prevalence is 3.8%, whereas in those above 60 years of age, it increases to 9.5%. In the United States, the estimated prevalence rate is 2.9%, with higher rates in men (4.2%) than in women (2.1%). When examining age-specific graphs of cardiovascular disease (CVD) death rates, there appears to be a notable gender disparity, which gradually reduces after menopause [3]. Cardiac mortality in women shows a significant increase around the age of 50, coinciding with menopause. This has led to the theory that the decline in estrogen levels during menopause might elevate the risk of CVD [4]. However, evidence from vital statistics challenges this assumption, suggesting that menopause itself does not directly increase the risk of cardiovascular disease. Instead, the narrowing of the gender gap in CVD appears to be driven by a decrease in incidence rates among men [2-5].

Estrogen and its role in cardiovascular health

Estrogen, primarily associated with the female reproductive system, exists in different forms, such as estrone, estradiol, and estriol. Estradiol is the most commonly used form of hormone replacement therapy (HRT) to relieve menopausal symptoms [11]. Estriol in HRT is being extensively researched, but it is still a subject of ongoing debate, and estrone is not used in HRT. These different types can be administered through various routes, such as oral, transdermal, creams, patches, vaginal inserts, or subdermal pellets. Each method is associated with its own pros and cons [12,13]. For instance, oral estrogen intake may increase the risk of blood clot formation due to activated protein C resistance and stimulate the liver to produce matrix metallic protease 9, affecting atherosclerotic plaque formation and rupture, leading to MI. On the other hand, transdermal estrogen will bypass the liver and reduce the risk of blood clot formation, but is not as effective as oral estrogen in postmenopausal women [14,15].

Estradiol (E2) interacts with various estrogen receptors, including ERα, ERβ, and GPR30, triggering both genomic and rapid non-genomic effects [16,17]. When E2 binds to ERα and ERβ receptors, it activates the genomic pathway and regulates gene transcription. Simultaneously, receptors rapidly activate nuclear transcription factors through the MAPK pathway. In cardiac tissue, these receptors are also found on the mitochondrial membrane, where their activation induces transcriptional changes in nuclear and mitochondrial genes, influencing mitochondrial function and cell survival, and providing cardioprotective effects [18]. Studies have shown that activating aldehyde dehydrogenase (ALDH) protects the heart from ischemic damage. Investigations into E2's cardioprotection via phosphoinositide 3-kinases (PI3K) signaling demonstrated that PI3K inhibition reduced cardioprotection and p-ALDH2 levels in female rat hearts after reperfusion injury [19].

Sex hormones, particularly estrogen, play a key role in this protection, as evidenced by the activation of estrogen receptor (ER) ERα, which reduces MI size and induces vasodilation and neovascularization. E2 also exerts cardioprotective effects through GPR30 activation on the mitochondrial surface. Moreover, it allows for increased calcium uptake by mitochondria before 1-methyl-4-phenyl-1,2,3,6-tetrahydropyridine (mPTP) opening, dependent on extracellular signal-regulated kinase (ERK) activation, as inhibiting ERK blocks the cardioprotective benefits [3]. Overall, E2 enhances mitochondrial structure and function, activating cardioprotective signaling pathways (PI3K/ERK1/2), inhibiting mPTP opening, and reducing reactive oxygen species (ROS) production by boosting potent antioxidants, SOD2, and hydrogen sulfide synthesis [18]. These findings highlight the multifaceted role of E2 in cardioprotection and its potential as a therapeutic target for ischemic heart disease.

Estrogen (E2) has significantly promoted vascularization and protected the heart from ischemic injury. Studies have demonstrated that E2 increases the incorporation of endothelial progenitor cells (EPCs) into areas of cardiac ischemic vascularization, providing protection against ischemic injury [19-21].

Hormone replacement therapy (HRT) and MI risk

References to menopause exist as far back as the 300s BC. In the 1800s, women who had silver-fork through menopausal symptoms injected themselves with bovine ovarian tissue to reduce sexual dysfunction. The 2022 Hormone Therapy Position Statement of The North American Menopause Society (NAMS) laid down clear guidelines on the use of HRT with detailed information about formulation, dosing, and routes of administration, with a clear mention of using progestogens to avoid endometrial overgrowth during the use of estrogen. The document further lists the reason for using HRT for Cardiovascular Disease and All-Cause Mortality by stating that for women within 10 years of the perimenopausal period, the benefits of hormone therapy exceed its risks, with fewer CVD episodes in younger women [22].

After the NAMS guidelines, several studies have emphasized the importance of individualized hormone replacement therapy in postmenopausal women, with consideration of factors including but not limited to therapy initiation, patient characteristics, and underlying illness. For instance, regarding HRT duration, using it for more than 60 days with estrogen-only or combined hormones demonstrated a significant risk reduction of acute MI [23]. On the other hand, transdermal estrogen has been suggested as a safer option in patients with a history of thrombotic events, stroke, and/or coronary artery disease. Short-term therapy provides protection against MI, but this protection reduces over the years as estrogen levels in the body deplete. A study showed that estrogen given for more than 60 months protects against the development of any cardiovascular disease if given within 10 years of menopause [22-25]. Unopposed estrogen usage for five years or more is also associated with a significant decrease in the incidence of the first MI. The mode of estrogen delivery in HRT also influences its impact on CVD prevention. Transdermal estrogen therapy provides a more sustained release and steady blood levels of estrogen compared to oral.

Initiating therapy within six years of menopause may gradually impact subclinical atherosclerosis more than starting placebo treatment 10 or more years after menopause. However, oestradiol's influence on cardiac measurements of atherosclerosis was inconclusive [23]. This suggests that HRT's impact on preventing coronary artery disease is influenced by treatment duration rather than the type of HRT used. While estrogen has demonstrated cardiovascular benefits in multiple research trials, the mode of administration and age of initiation are still topics of discussion. Individual evaluation, considering risk factors and comorbidities, is necessary as responses to HRT can vary among women [24]. Various studies are ongoing on personalizing hormonal-based therapy based on cardiovascular risk each and every time since menopause. This research is essential for maximizing benefits, minimizing risk, and overcoming personal risk factors. While these studies shed light on the potential benefits of HRT in preventing CVD and mortality in women, further research is needed to better understand and optimize its use.

It is being observed in various studies that menopause may increase inflammation-related responses to antiestrogen therapy as it protects against endothelial injury, platelet aggregation, and plaque initiation, eventually reducing the risk of atherosclerosis development. Along with the initiation, the duration of estrogen therapy also plays a crucial role in administration, resembling the physiological environment [25]. In conclusion, individualized HRT decisions, considering the timing of initiation, dose, duration, formulation, and route of administration, are crucial for maximizing cardiovascular benefits and minimizing risks. Starting estrogen therapy early after menopause and utilizing transdermal delivery with appropriate dosages may offer cardioprotective effects, but personalized approaches based on each woman's risk factors and health status are essential.

Modifying factors and interactions

Age and menopausal status act as effect modifiers in the relationship between estrogen use and MI. With advancing age, the risk of MI increases in both women and men due to factors like elevated blood pressure, cholesterol levels, diabetes, and older age itself. On the other hand, menopausal status influences the clinical consequences of MI in women, with protection against atherosclerosis observed in premenopausal women. Hormone disturbances and disorders in women can lead to atherosclerosis and MI at a younger age. It is challenging to say that estrogen is increasing the risk of coronary artery disease in men [26]. Hence, opening a wide potential for research in this field can be beneficial.

The impact of estrogen may differ based on age, with early initiation of HRT potentially offering cardiovascular benefits, while starting it later could increase the risk of cardiovascular diseases. Hypertension and smoking can also influence estrogen's effects; long-term estrogen use in patients with hypertension may exacerbate the condition, and nicotine's estrogen-inhibiting activity can impact the efficiency of exogenous estrogen in postmenopausal women. Obesity, which affects circulating estrogen levels and HRT, may have long-term adverse effects on cardiovascular health, including hypertension [27]. Considering these risk factors is crucial when prescribing estrogen therapy to ensure personalized treatment plans for improved cardiovascular outcomes.

Genetic and epigenetic factors play a significant role in influencing MI risk and the effects of estrogen use. Estrogen influences gene expression through genomic and non-genomic mechanisms, making its effects complex and individual-specific. Genetic factors, such as a family history of MI, can impact the efficacy of estrogen as HRT and increase the risk of MI in women. Inherited genetic variants may also affect lipid metabolism and cholesterol levels, influencing plaque accumulation and atherosclerosis development, a major cause of MI. Epigenetic processes also mediate estrogen's effects, with studies showing that estrogen utilization and cognitive functions like memory enhancement are dependent on epigenetic mechanisms [27]. Understanding the interplay between genetic and epigenetic factors with estrogen use is vital for developing personalized approaches to estrogen therapy and optimizing its benefits while minimizing risks related to cardiovascular health and memory enhancement. Further research in this area will be essential to enhance our understanding and tailor estrogen therapy for individual patients.

Controversies and conflicting evidence

One of the major controversies is related to the timing of estrogen initiation. As discussed above, several studies suggest that early initiation of HRT, particularly within the first 10 years after menopause, reduces the incidence of cardiovascular diseases along with protecting against atherosclerosis and MI. However, other studies have demonstrated that early use of estrogen for five years is protective, but after stopping estrogen and the levels dropping in the blood, the protective effect wanes. This creates a scope of long-term estrogen therapy contraindicated by several other studies that state that long-term use of estrogen is harmful.

Another conflicting evidence that has been a topic of debate is the mode of estrogen administration. It is generally established that any medication that bypasses past metabolism is considered safe, including transdermal estrogen in the form of patches or gels. Transdermal estrogen has potentially been used in patients with a history of thrombotic events to improve cardiovascular outcomes. However, evidence exists describing the potential disadvantages of transdermal estrogen and establishing the need for more research for optimal mode of administration. Another aspect is the duration of estrogen therapy. Short-term use of estrogen has been associated with cardioprotective effects, but long-term use may lead to decreased serum estrogen levels, potentially diminishing its protective benefits over time.

The mode, duration, and timing of estrogen are interlinked, creating conflicting evidence in various research trials. Hence, further evidence is needed to consider mild doses and timing of estrogen in postmenopausal women. There have been conflicting studies on the use of HRT in premenopausal women, mostly because of its risk of causing thrombotic episodes in premenopausal women. There needs to be a study of estrogen use age in premenopausal women with hysterectomy and bilateral oophorectomy.

Furthermore, the interplay of genetic and epigenetic factors with estrogen use adds complexity to understanding its effects on cardiovascular health. Genetic variations and family history may influence the response to estrogen therapy and the risk of adverse outcomes. It is essential to consider individual patient characteristics, such as age, menopausal status, cardiovascular risk factors, and genetic predispositions, when making decisions about estrogen therapy. More research is needed on personalized treatment approaches to optimize cardiovascular outcomes while ensuring patient safety.

Current guidelines and recommendations

The current guidelines and recommendations regarding the use of estrogen therapy are based on evidence from clinical trials and expert consensus. There is no solid guideline or regimen recommended till today. Some guidelines suggest that HRT may be considered for women experiencing bothersome menopausal symptoms, especially during the early menopausal transition. Early initiation of HRT, within the first 10 years after menopause, may offer potential cardiovascular benefits, including protection against atherosclerosis [28,29].

Transdermal estrogen delivery systems, such as patches or gels, may be preferred over oral estrogen due to their potentially better safety profile. Transdermal estrogen bypasses the liver's first-pass metabolism, potentially reducing the risk of thrombotic events. However, further research is needed to fully establish the optimal mode of administration. Short-term use of estrogen may provide cardioprotective effects, but long-term use may lead to decreased serum estrogen levels and diminish its protective benefits over time. Healthcare providers should regularly reassess the need for ongoing therapy and consider the potential risks and benefits in each individual case [30,31].

Personalized treatment approaches are essential in estrogen therapy. Individual patient characteristics, including age, menopausal status, cardiovascular risk factors, and genetic predispositions, should be considered when making decisions about HRT. The use of estrogen should be tailored to each patient's specific needs and risks.

It's important to note that guidelines and recommendations may vary among different medical organizations and may be updated periodically based on new research and evidence. Therefore, healthcare providers should stay up to date with the latest guidelines and consider the unique characteristics of each patient when prescribing estrogen therapy.

Future directions and research gaps 

Research predicting individual responses to estrogen therapy by identifying biomarkers and genetic factors will enable healthcare providers to tailor treatment plans for better outcomes and minimize potential risk factors. Further investigation to compare the cardiovascular outcomes between transdermal and oral administration can help in optimizing the mode of estrogen delivery as well as having a better understanding of potential adverse effects. Research can also focus on identifying the most effective and safe combination with estrogen for women with specific cardiovascular risk factors. 

Understanding the potential benefits and risks associated with estrogen and cognitive function, including but not limited to memory loss and risk of dementia, can guide treatment decisions for women with cardiovascular and psychiatric risk factors. Studies/research can take into consideration postmenopausal women with psychiatric conditions such as depression, coma, anxiety, and bipolar diseases and estrogen usage in these women and its potential concern for cardiovascular benefit versus its effect on cognitive function. It can also focus on the effects of quality of life, evaluating the impact of treatment on symptom relief and overall well-being in patient-centered care. 

Research of non-hormonal therapies for the treatment of menopausal symptoms versus estrogen therapy can benefit. Non-hormonal therapies can include dietary adjustments for instant milk-based products for soothing cramping pain. Physical activity, stress management for reducing symptoms of hot flashes, and sleep hygiene can help some women reduce the need for estrogen therapy. Research investigating the possibility of individualized therapies such as vitamins, oxidants, and herbal products, which help in improving menopausal symptoms as well as have a cardio-protective effect, can also be considered. 

Researching novel hormone treatments can also be considered. For the research on mind-yoga therapy, such as yoga, meditation, and mindfulness-based stress reduction, can be beneficial for heart and general well-being in postmenopausal women. Integrating conventional medical treatments with complementary and alternative therapies in a holistic approach may offer comprehensive care for menopausal women's cardiovascular health. 

The effectiveness of long-term estrogen therapy in postmenopausal women requires further study. Large-scale randomized control trials with extended follow-up periods need to evaluate the possible hazards and advantages of estrogen therapy in various patient populations. Targets and medicines can be created by using genomic and non-genomic processes to precisely understand how estrogen protects the heart. This will lead to Major breakthroughs and help us establish the initiation of medicine, dosage, and route in a better way rather than personalizing it as per current guidelines and physicians' practice. 

In conclusion, future research should address the existing gaps in our understanding of estrogen therapy and explore emerging therapies and alternative approaches to enhance cardiovascular health in menopausal women. By advancing our knowledge in these areas, healthcare providers can offer more personalized and effective interventions for this patient population. 

## Conclusions

The cardiovascular effects of estrogen require a careful balancing act between possible advantages, such as enhanced lipid profiles and vascular function, and possible concerns, like increased thrombotic risk. Estrogen has cardioprotective properties in premenopausal women, but there is a need for further research to show its effect on postmenopausal women and men. 

Because MI is a multifaceted disorder influenced by a person's lifestyle, genetics, and environment, estrogen is only one piece of the prevention puzzle. For the prevention of MI, a holistic approach to cardiovascular health is essential, encompassing lifestyle modifications, risk management, and individualized medicinal therapies.

Utilizing hormone replacement treatment (HRT) for postmenopausal women should be done cautiously, taking into account the unique patient characteristics and possible hazards. Healthcare professionals should be involved in HRT decision-making and take cardiovascular risk and general health into consideration.

## References

[1] Yang XP, Reckelhoff JF (2011). Estrogen, hormonal replacement therapy and cardiovascular disease. Curr Opin Nephrol Hypertens.

[2] Hammes SR, Levin ER (2019). Impact of estrogens in males and androgens in females. J Clin Invest.

[3] Khan MA, Hashim MJ, Mustafa H (2020). Global epidemiology of ischemic heart disease: results from the Global Burden of Disease Study. Cureus.

[4] Salari N, Morddarvanjoghi F, Abdolmaleki A (2023). The global prevalence of myocardial infarction: a systematic review and meta-analysis. BMC Cardiovasc Disord.

[5] Sybil LC, Johannes CB (1999). The epidemiology of cardiovascular disease in postmenopausal women. J Clin Endocrinol Metab.

[6] Gaziano TA, Bitton A, Anand S, Abrahams-Gessel S, Murphy A (2010). Growing epidemic of coronary heart disease in low- and middle-income countries. Curr Probl Cardiol.

[7] Nabel EG, Braunwald E (2012). A tale of coronary artery disease and myocardial infarction. N Engl J Med.

[8] Brieger D, Fox KAA, FitzGerald G (2009). Predicting freedom from clinical events in non-ST-elevation acute coronary syndromes. The global registry of acute coronary events. Heart.

[9] Roe MT, Messenger JC, Weintraub WS (2010). Treatments, trends, and outcomes of acute myocardial infarction and percutaneous coronary intervention. J Am Coll Cardiol.

[10] Berry JD, Dyer A, Cai X (2012). Lifetime risks of cardiovascular disease. N Engl J Med.

[11] Zakharova MY, Meyer RM, Brandy KR (2011). Risk factors for heart attack, stroke, and venous thrombosis associated with hormonal contraceptive use. Clin Appl Thromb Hemost.

[12] Hulley S, Grady D, Bush T, Furberg C, Herrington D, Riggs B, Vittinghoff E (1998). Randomized trial of estrogen plus progestin for secondary prevention of coronary heart disease in postmenopausal women. Heart and Estrogen/progestin Replacement Study (HERS) Research Group. JAMA.

[13] Grady D, Herrington D, Bittner V (2002). Cardiovascular disease outcomes during 6.8 years of hormone therapy: Heart and Estrogen/progestin Replacement Study follow-up (HERS II). JAMA.

[14] Delgado BJ, Lopez-Ojeda W (2023). Estrogen.

[15] Harper-Harrison G, Shanahan MM (2023). Hormone replacement therapy. https://www.ncbi.nlm.nih.gov/books/NBK493191/.

[16] Shufelt CL, Merz CN, Prentice RL (2014). Hormone therapy dose, formulation, route of delivery, and risk of cardiovascular events in women: findings from the Women's Health Initiative Observational Study. Menopause.

[17] Bopassa JC, Eghbali M, Toro L, Stefani E (2010). A novel estrogen receptor GPER inhibits mitochondria permeability transition pore opening and protects the heart against ischemia-reperfusion injury. Am J Physiol Heart Circ Physiol.

[18] Babiker FA, De Windt LJ, van Eickels M (2004). 17beta-estradiol antagonizes cardiomyocyte hypertrophy by autocrine/paracrine stimulation of a guanylyl cyclase A receptor-cyclic guanosine monophosphate-dependent protein kinase pathway. Circulation.

[19] Lagranha CJ, Deschamps A, Aponte A, Steenbergen C, Murphy E (2010). Sex differences in the phosphorylation of mitochondrial proteins result in reduced production of reactive oxygen species and cardioprotection in females. Circ Res.

[20] Iorga A, Cunningham CM, Moazeni S, Ruffenach G, Umar S, Eghbali M (2017). The protective role of estrogen and estrogen receptors in cardiovascular disease and the controversial use of estrogen therapy. Biol Sex Differ.

[21] Lenhart PM, Broselid S, Barrick CJ, Leeb-Lundberg LM, Caron KM (2013). G-protein-coupled receptor 30 interacts with receptor activity-modifying protein 3 and confers sex-dependent cardioprotection. J Mol Endocrinol.

[22] Chrisandra L. Shufelt, Vivien Brown, Janet S (2022). The 2022 hormone therapy position statement of the North American Menopause Society. Menopause.

[23] Xiang D, Liu Y, Zhou S, Zhou E, Wang Y (2021). Protective effects of estrogen on cardiovascular disease mediated by oxidative stress. Oxid Med Cell Longev.

[24] Hodis HN, Mack WJ, Henderson VW (2016). Vascular effects of early versus late postmenopausal treatment with estradiol. N Engl J Med.

[25] Holly LT, Hodis NH (2008). Assessing benefits and risks of hormone therapy in 2008: new evidence, especially with regard to the heart. Cleve Clin J Med.

[26] Frick KM, Zhao Z, Fan L (2011). The epigenetics of estrogen: epigenetic regulation of hormone-induced memory enhancement. Epigenetics.

[27] Jeong SM, Yoo JE, Jeon KH, Han K, Lee H, Lee DY, Shin DW (2023). Associations of reproductive factors with incidence of myocardial infarction and ischemic stroke in postmenopausal women: a cohort study. BMC Med.

[28] Ruberti OM, Rodrigues B (2020). Estrogen deprivation and myocardial infarction: role of aerobic exercise training, inflammation and metabolomics. Curr Cardiol Rev.

[29] Carr BR, Ory H (1997). Estrogen and progestin components of oral contraceptives: relationship to vascular disease. Int J Reprod Contracept.

[30] Lewis MA, Heinemann LA, Spitzer WO (2003). The use of oral contraceptives and the occurrence of acute myocardial infarction in young women. Europ Heart J.

[31] Rosenberg L, Palmer JR, Sands MI (1997). Modern oral contraceptives and cardiovascular disease. Am J Obstet Gynecol.

